# Predictors of parental mediation in teenagers’ internet use: a cross-sectional study of female caregivers in Lahore, Pakistan

**DOI:** 10.1186/s12889-021-10349-z

**Published:** 2021-02-08

**Authors:** Sarosh Iqbal, Rubeena Zakar, Florian Fischer

**Affiliations:** 1 Institute of Social and Cultural Studies, University of the Punjab, Lahore, Pakistan; 2 Department of Public Health, Institute of Social and Cultural Studies, University of the Punjab, Lahore, Pakistan; 3 Institute of Public Health, Charité – Universitätsmedizin Berlin, Berlin, Germany; 4 Institute of Gerontological Health Services and Nursing Research, Ravensburg-Weingarten University of Applied Sciences, Weingarten, Germany

**Keywords:** Teen, Children, Online, Internet addiction, Risk, Resilience

## Abstract

**Background:**

The internet has become the most widely used medium among teenagers, who spend much of their time online, which raises parental concerns. Notwithstanding teens’ increased internet use and exposure to online risks, little is yet known about parental internet mediation in local settings. The present research aimed to assess the various dimensions of parental mediation to regulate teens’ use of the internet and their predictors.

**Methods:**

A cross-sectional survey was conducted in the district of Lahore, Pakistan, among mothers/female caregivers of teens (aged 13–19 years). Only women were interviewed because they are more frequently engaged as primary caregivers than fathers or male caregivers. Furthermore, only qualified and working mothers from the top two professions among women, i.e. academia and medicine, were interviewed. A stratified random sampling technique was adopted, and 347 mothers were interviewed using face-to-face interviews at 11 universities and 11 hospitals/medical colleges. Data were entered and analysed using descriptive, bivariate and multivariate logistic regression analyses.

**Results:**

The findings highlighted that more than 65% of respondents applied highly active mediation of internet safety, around 60% used highly active co-use mediation and more than 56% applied restrictive mediation. In addition, 36% of respondents monitored and 15.3% technically mediated to regulate their teens’ use of the internet. The results of the multivariate logistic regression revealed that the majority of respondents were more inclined to adopt active internet safety mediation if they had teens aged 16–19 years, with medium internet addiction, possessed good digital skills, felt confident about their teens’ coping appraisal to perform online protection, and considered their teens to have high self-esteem and resilience.

**Conclusions:**

This research found that parental internet mediation is a multifaceted concept used to regulate teens’ online activity and enhance a resilient approach to reduce the risks associated with use of the internet. The researchers recommend developing parental guidelines, e-safety resource material, local support networks and community programmes to educate parents, teachers and teens in order to raise awareness and promote resilient pathways amongst teens.

**Supplementary Information:**

The online version contains supplementary material available at 10.1186/s12889-021-10349-z.

## Background

The internet has become the most widely used medium among the young generation in today’s media- and technology-rich environment, particularly among teenagers, commonly known as ‘teens’. Even at the beginning of the widespread usage of the internet, teens used it for more hours than adults [1]. Nowadays, teens have been born and raised in a digital era and, hence, are also recognised as ‘digital natives’ [2]. Online virtual environments stimulate teens’ self-presentation and identity experiments, particularly through the sharing of their self-created content, posts and pictures online [3]. For this reason, they are considered more digitally literate than their parents – leading to a generation gap [4–6]. The increased use of the internet among teens, their concerns about online identity and privacy, and their strong association with peers, alongside reduced communication with parents, enhance their susceptibility to online risks [7].

Teenage years are coupled with developmental changes. Teens mostly devote their time online to using self-selected devices for recreational and social activities without any parental supervision [8]. These unsupervised activities have a long-lasting impact on them; therefore, parents apply multiple dimensions of mediation, particularly to promote positive outcomes amongst teens. Internet use has mounted over the past two decades with free web browsing, social networking, online shopping, gaming and instant messaging. Furthermore, the introduction of smartphones and multiple ‘apps’ has also fuelled internet use [9]. The internet provides numerous benefits in the areas of information, edutainment and socialisation; nonetheless, it also exposes users to a unique set of online risks, such as privacy invasion, cyberbullying and exposure to violent, hateful or inappropriate material or contacts [5]. Moreover, the effects of online risks, such as pornography, on teens and the adverse impact on youths’ self-esteem is a matter for concern [10]. Therefore, high sensitivity and concern among parents about their teens’ risks related to online addiction and victimisation are needed in order to protect teens from the negative aspects of internet use and to avoid harm [9].

Given this context, the concept of parental mediation (PM) has emerged. Parental internet mediation acknowledges that parents actively manage and regulate their children’s internet use [11], while mitigating its negative effects amongst teens [12]. The notion of PM originated primarily in media studies, especially in the areas of television and video games, to comprehend the effects of media content on teens’ or children’s behaviour [13]. Researchers have demonstrated that young audiences adopt certain behaviours that are presented on television and in video games unless parents mediate [14]. Hence, parental involvement encourages the potential for positive outcomes, while also effectively neutralising the negative effects of the internet [15].

Previous studies on television and video games have categorised PM into three dimensions: instructive or active, restrictive and co-use mediation [16–18]. Furthermore, with the evolution of the internet and digital devices, for example, smartphones and tablets, different researchers have strengthened and refined the concept of PM over a period of time. Livingstone et al. [19] recently recognised that digital devices and the internet, being more technologically complex, personalised and portable than previous technology, were difficult for parents to manage. Hence, five dimensions of parental internet mediation were developed, keeping in view the specific attributes of the internet. These are: 1) active co-use or instructive mediation, where parents encourage, share and discuss mutually; 2) active mediation of internet safety, where parents guide teens towards safer online practices; 3) restrictive mediation, where parents set rules and regulations; 4) monitoring, where parents check the record available afterwards; and 5) technical mediation, where parents use software or control mechanisms to restrict, filter or monitor online activities [20].

Previous research suggests that parental preferences for applying these various dimensions of PM are subject to multiple predictors, such as the teens’ online addiction and parents’ own characteristics, including education, income and digital skills [21]. Moreover, parents’ beliefs about risk and response appraisal, as well as their effect on teens, also determine the various dimensions of PM.

Giving due importance to parental beliefs and inputs, the theoretical foundations of this research lie in Protection Motivation Theory (PMT) to aid in understanding PM-related predictors. The PMT postulates that one’s intention to adopt protective behaviour is linked to how individuals process threats and cope with adverse circumstances [22]. Under the ambit of this research, PMT suggests that parents’ own perceptions of threat and coping appraisal could be the predictors of PM. Severity indicates the seriousness of an online risk in threat appraisal, while susceptibility refers to vulnerability towards these risks. Furthermore, threat appraisal also considers the teens’ (excessive) internet use. Parents, for instance, who found online addiction among their teens, perceived the online risks to be more severe and believed their teens to be more susceptible to them applied mediation. Under coping appraisal, response efficacy denotes effectiveness in preventing risks, while self-efficacy indicates the individual’s ability to achieve optimal online safety behaviour. Parents mediate more often, for example, if they believe that their involvement enables teens to manage the online risks effectively and adopt online protection behaviour. Coping appraisal also highlights parents’ own digital skills, which help them to evaluate their teens’ responses and self-efficacy. Hence, taken altogether, PMT proposes that PM could be considered as self-protective behaviour against adversity and online risks [23, 24]. This adversity could be overcome through the teens’ higher self-esteem and resilience. Resilience is defined here as a strength-based and positive outcome in the face of online risks or challenges [25].

Parental internet mediation is the concern of all parents and societies with the widespread use of digital technologies, regardless of background or culture. However, although there are multiple PM-related studies available for western societies and cultures [26], there is a dearth of comparable literature for eastern societies and cultures, such as Pakistan. The latter ranks in the top 10 among countries within the Asian region regarding digital growth [27]. There are currently more than 44 million internet users in the country [28]. Among them, a majority of young people and teens surf the internet for a minimum of 2 h a day, largely gaining access on tablets and smartphones [29]. There is a substantial cultural difference between eastern (Asian) and western parenting practices [30]. Moreover, the notion of parental internet mediation is quite new in the developing country of Pakistan. Notwithstanding the teens’ increased internet use or addiction and their exposure to online risks, little is yet known about parental internet mediation or the factors influencing it in local settings.

**Fig. 1:**
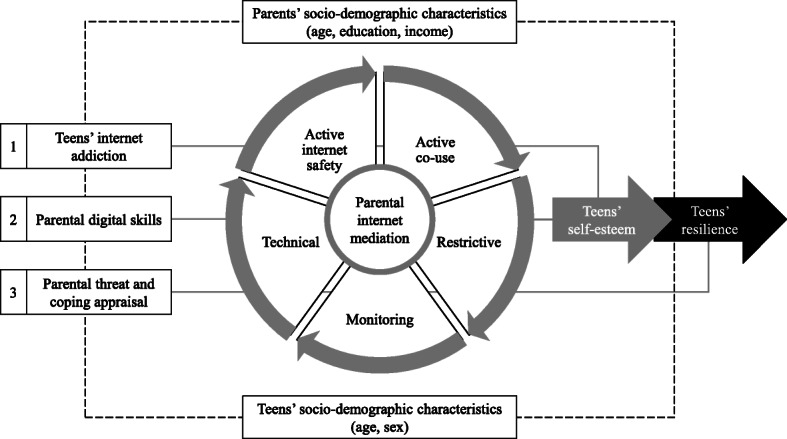
Theoretical framework

Given the context above, this research is an attempt to fill the gap in the existing literature and seeks to understand the varied dimensions of PM to regulate the teens’ use of the internet and their predictors in the district of Lahore (Pakistan), as illustrated in Fig. 1. These predictors include socio-demographic and teen-related characteristics, the teens’ internet addiction, parents’ own digital skills, parents’ assessments of threat and coping appraisals, as well as the effects of PM among teens, particularly in nurturing self-esteem and resilience.

## Methods

### Research setting and participants

A cross-sectional survey was conducted in Lahore, a district in Pakistan. Lahore District is the capital of Punjab province, with 11 million inhabitants. It is the second largest and most populated urban district in Pakistan [31]. Almost one quarter of the population is below 19 years of age [32].

Parents or primary caregivers of teens aged 13–19 years were included in the study. Only mothers or female caregivers were interviewed, because they are more frequently involved as primary caregivers than fathers or male caregivers [33]. Furthermore, the study focused only on qualified and working mothers from the top two professions chosen by women, i.e. academia and medicine. This focus was chosen because, with higher education, serving professionals and those belonging to the middle class or having high socio-economic status are more aware of internet-related benefits and risks and are, therefore, more likely to apply mediation [34–36]. Learning from previous studies [34–36], this research narrowed its focus only to the mothers belonging to middle to high socio-economic status due to several facts. Firstly, teens from the middle to high social class use the internet more often on a range of devices compared to the low social class [37]. Secondly, parents from the middle to high social class and education have more awareness about online risks, thus, report more online harm to their children/teens and mediate more often [37]. Thirdly, literature also recommends that PM depends upon the family’s socio-economic status, where parents from middle to high status raise their children in digitally rich environments and home ecologies, which determine their quantity and quality of internet use, as well as parents’ confidence in mediation [38]. Consequently, associate and assistant professors and lecturers from academic universities were interviewed, while lady doctors and head nurses were interviewed from hospitals and attached medical colleges in the district of Lahore.

### Sampling strategy

The Cochran formula of $n=\frac{z_{\alpha /2}^2\bullet p\bullet \left(1-p\right)}{d^2}$ was used to calculate the sample size [39], assuming z = 95% significance level, α = 95% confidence interval, *p* = 18% population proportion using the internet [28], d = 5% absolute precision and 1.5 design effect. This formula led to a sample size of 340, which was also selected by considering the minimum number required for the central limit theorem, a key principle of statistics for ensuring the cost-effectiveness of population surveys [40].

### Development of the questionnaire

A closed-ended and precoded interview schedule was prepared to address the research objectives (Supplementary Appendix 1). This interview schedule was pretested before finalisation and going into the field. Pretesting helped to determine the effectiveness of the interview schedule, particularly related to the language, wording, order, format of questions and analytical approach. Based on the findings of the pretesting, the interview schedule was finalised. Furthermore, the validity and reliability of the interview schedule were also measured using Cronbach’s alpha, which was found to be satisfactory.

#### Outcome variable

Parental internet mediation was the outcome variable. It was assessed in five dimensions, measured through selecting multiple situations on closed-ended responses (yes/no), which is consistent with previous similar studies [20]. Firstly, the respondents’ active co-use mediation was assessed, such as talking about the teens’ online activities, sharing activities together, sitting alongside or staying nearby during internet use, or encouraging teens to learn and explore things online. Secondly, restrictive mediation was measured by asking whether parents either restrict their teens or give permission for them to own social media profiles, share personal information, use instant messaging, watch and download music/films/videos or upload videos/photos online. Thirdly, the respondents’ monitoring was assessed in terms of checking social media profiles, friends lists and account messages, and visiting websites after the teens’ internet use. Fourthly, the respondents’ technical mediation was measured by their use of parental control mechanisms to filter, block or track websites and their use of software to limit or protect teens from viruses or spam mails. Lastly, the respondents’ active mediation of internet safety was assessed in terms of helping or suggesting safety measures to their teens or having supported their teens in the past when they were bothered online. A mean score was used to dichotomise all 5 PM dimensions into high and low mediation after computing all the variables above.

#### Independent variables

The independent variables consisted of socio-demographic characteristics and other predictors, as mentioned in the theoretical framework. Socio-demographic variables included the respondents’ age in years (31–40, 41–50, 51–60), marital status (married, divorced, separated, widowed), monthly income in PKR (up to 50,000; > 50,000–100,000; > 100,000–150,000; > 150,000–200,000; > 200,000), the teens’ age in years (13–15, 16–17, 18–19) and gender (boy, girl). Other predictors included internet addiction, digital skills, threat and coping appraisal, and the teens’ self-esteem and resilience.

The respondents’ perception of the teens’ addictive behaviour was measured through selecting five situations (yes/no), consistent with the literature [41], in which, due to the internet, teens: 1) do not eat or sleep; 2) spend less time with family and friends; 3) are caught surfing when not interested; 4) feel bothered when not online; and 5) unsuccessfully tried to spend less time on the internet. The categories of high, medium and low internet addiction were constructed after computing the index.

Another key variable was the respondents’ own understanding of multiple digital skills [42], such as comparing or bookmarking websites, changing filters or privacy settings, blocking messages or unwanted pop-ups/adverts or spam/junk mails, and deleting the record of websites visited. A closed-ended response on multiple situations was recorded (yes/no). This variable was dichotomised into good or weak digital skills after computing the index.

An important predictor is the respondents’ threat appraisal, which was measured in terms of the severity of the threat and the teens’ susceptibility to potential online risks. Initially, female caregivers were asked about how serious they considered the online risks to teens (serious vs. not serious), such as online threats, receiving hate-based or sexual remarks, someone pretending to be a teen, publishing personal information, videos, pictures or negative comments about teens with bad intentions, or being infected with a computer virus [43]. The respondents were subsequently asked how likely they felt it was that the online risks mentioned above, excluding the virus, might happen to teens (likely vs. not likely) [43]. The variables were dichotomised after computing the indices above.

The respondents’ coping appraisal was also measured in terms of the two elements of the teens’ response-efficacy and self-efficacy to ensure online safety. The respondents’ perception of their teens’ response-efficacy was measured against a 6-item list (agree vs. disagree): use of a nickname to conceal their identity and personal information, providing inaccurate information for privacy protection, limiting access to only friends/family, avoiding contact with online strangers, being aware of whom to talk to for online safety advice, and believing that they could receive help from parents and teachers in the form of good advice [43]. Similarly, the respondents’ opinion about their teens’ response-efficacy was assessed against the same 6-item list (likely vs. not likely) [43]. Dichotomous variables were constructed after computing the indices above.

In order to determine the respondents’ opinions of their teens’ self-esteem, Rosenberg’s [44] 10-item scale was used (yes/no), which is widely recognised as a reliable and valid instrument to measure self-esteem in multiple settings including Pakistan [45, 46]. This scale includes both positive qualities (feels satisfied, worthy, capable, has a positive attitude and good behaviour) and negative qualities (feels useless, bad, a failure, nothing to be proud of and wants to earn more respect). It was dichotomised into high vs. low self-esteem after computation of the index. Furthermore, the research measured the respondents’ opinions of their teens’ resilience using the 12-item child and youth resilience measure, which was explicitly developed for parents/caregivers [47]. A closed-ended response to multiple situations was recorded (yes/no). These items about the teens’ resilient behaviour include living with likeable people, learning useful things, completing all tasks, considering education and institutions important, being aware of how to fix things and seeking help, liking community-related celebrations/festivals and their treatment, and always getting support from friends and family during difficult times. The variable was dichotomised into high vs low resilience after computing an index. Details of the above indices are provided in Supplementary Appendix 2.

### Data collection

**Fig. 2:**
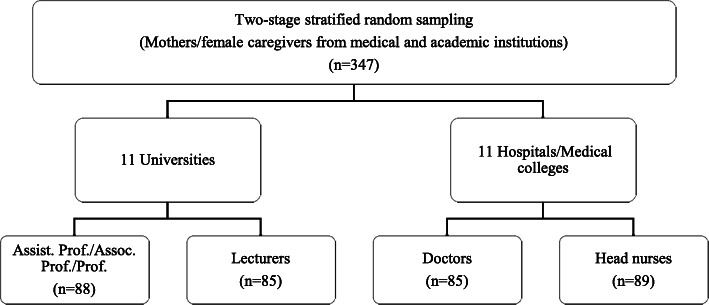
Two-stage stratified random sampling for survey

The survey was conducted during April and May 2018 using two-stage stratified random sampling, during the first stage of which 11 universities and 11 hospitals/medical colleges were randomly selected, based on district-specific lists of academic universities and hospitals/medical colleges. These lists were obtained from the Punjab Higher Education Department for academic universities and the Pakistan Medical and Dental Council for hospitals/medical colleges. In the second stage, eligible respondents, i.e. mothers/female caregivers who had teens in the age range of 13 to 19 years, were randomly selected for the survey. The last-birthday method was applied when one eligible respondent had more than one teen [48]. Thus, a total of 347 respondents (mothers/female caregivers) were interviewed, as illustrated in Fig. 2, which presents the break-down of the stratified sampling.

### Data entry and statistical analysis

EpiData software was used for data entry. After this was complete, the data were exported and analysed in SPSS version 21 to present univariate descriptive, bivariable and multivariate logistic regression analyses. Reliability and internal consistency of all measures were calculated using Cronbach’s alpha, which was found to be acceptable between 0.72 and 0.86. Cross-tabulations were calculated, along with chi-square as a test of association, where *p*-values < 0.05 showed statistical significance. Odds ratios (OR) with 95% confidence intervals (CI) were calculated in bivariable logistics regression. The predictors showing significance with a p-value < 0.05 during bivariable analysis were further included in the regression modelling. Thus, five bivariate logistic regression models, with each of the PM dimensions, were calculated to identify the unadjusted effect of predictors on the outcome variables. Adjusted odds ratios (AOR) with 95% CI were also determined during the multivariate analysis.

## Results

### Socio-demographic characteristics

The respondents’ mean age was 47 years (SD + 5.44), with the majority (61.7%) belonging to the age group 41–50 years. Most women were married (93.4%), and had a monthly income between 51,000 and 100,000 Pakistani rupees (PKR) (45.8%). The mean age of the teens was 16 years (SD + 2.05) (Table 1).

**Table 1:** Socio-demographic characteristics of respondents (caregivers of teenagers) (*n* = 347)

Characteristics	***n***	%
**Respondents’ age**		
Median (*range*)	47.0 (31–60)	
M (*SD*)	47.13 (5.44)	
31–40 years	48	13.8
41–50 years	214	61.7
51–60 years	85	24.5
**Respondents’ marital status**		
Married	324	93.4
Separated	7	2.0
Divorced	7	2.0
Widowed	9	2.6
**Respondents’ monthly income (in PKR)**		
Up to 50,000	45	13.0
> 50,000–100,000	159	45.8
> 100,000–150,000	70	20.2
> 150,000–200,000	34	9.8
> 200,000	39	11.2
**Age of teens**		
Median (*range*)	16.0 (13–19)	
M (*SD*)	16.0 (2.1)	
13–15 years	145	41.8
16–17 years	97	28.0
18–19 years	105	30.3
**Sex of teens**		
Teen boys	183	52.7
Teen girls	164	47.3
**Respondents’ digital skills**		
Good skills	226	65.1
Weak skills	121	34.9
**Experience of teens’ internet addiction**		
No addiction	74	21.3
Medium addiction	69	19.9
High addiction	204	58.8
**Threat appraisal**		
**Severity of online risks**		
Serious	311	89.6
Not serious	36	10.4
**Susceptibility to online risks**		
Likely	208	59.9
Not likely	139	40.1
**Coping appraisal**		
**Response efficacy to perform online protection**		
Agree	213	61.4
Disagree	134	38.6
**Self-efficacy to perform online protection**		
Likely	191	55.0
Not likely	156	45.0
**Teens’ self-esteem**		
High self-esteem	206	59.4
Low self-esteem	141	40.6
**Teens’ resilience**		
High resilience	248	71.5
Low resilience	99	28.5

### Characteristics of parental internet mediation and its influencing factors

**Fig. 3:**
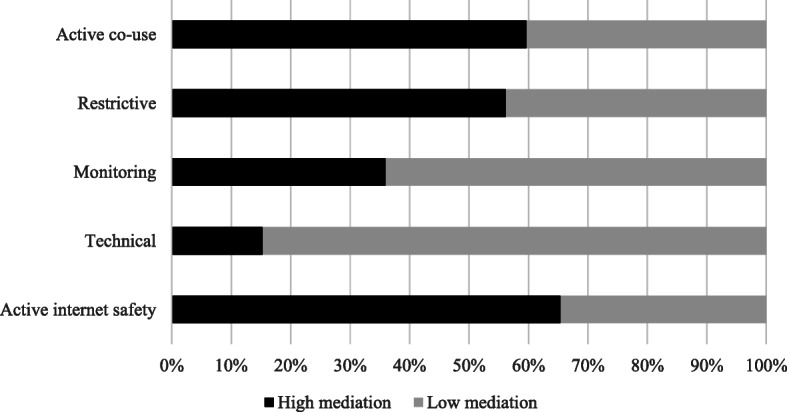
Parental perception of applying five dimensions of internet mediation

Figure 3 shows that more than 65% of the respondents applied highly active mediation of internet safety, around 60% applied highly active co-use mediation and more than 56% adopted restrictive mediation to regulate their teens’ use of the internet. About 36% of the respondents monitored their teens and only 15.3% applied technical mediation.

Table 1 highlights the predictors of parental internet mediation. The results show that most respondents (65.1%) had good digital skills. A large number reported high internet addiction amongst their teens (58.8%); only 21.3% confirmed no internet addiction among their teens. The findings reveal that 89.6 and 59.9% of the respondents reported severe online risks and susceptibility of teens, respectively. A large proportion was informed about response efficacy (61.4%) and self-efficacy (55.0%) to adopt online safety measures against risks. Lastly, the results indicate that respondents found high self-esteem (59.4%) and resilience (71.5%) among their teens.

### Bivariate logistic regression

The results of the bivariate logistic regression (Table 2) indicate that active mediation of internet safety was significantly associated with the teens’ age, internet addiction, digital skills, severity of online risks, self-esteem and resilience. Furthermore, 8 out of the overall 12 predictors included in the bivariate logistic regression model showed significant associations with the active mediation of internet safety. Particularly good digital skills among the respondents (OR = 4.05, 95% CI: 2.52–6.49) and judging the severity of online risks as serious (OR = 2.99, 95% CI: 1.48–6.06) showed strong associations with active mediation of internet safety. Technical mediation was only significantly associated with susceptibility to online risks.

**Table 2:** Bivariate logistics regression of parental internet mediation with various socio-demographic and PM predictors

Characteristics	Active co-use			Restrictive			Monitoring			Technical			Active internet safety		
	OR	95% CI	***p***-value	OR	95% CI	***p***-value	OR	95% CI	***p***-value	OR	95% CI	***p***-value	OR	95% CI	***p***-value
**Respondents’ age**															
31–40 years	1			1			1			1			1		
41–50 years	0.47	0.23–0.99	0.04	1.07	0.57–2.01	0.83	2.21	1.07–4.58	0.03	1.46	0.58–3.69	0.42	1.09	0.56–2.13	0.78
51–60 years	0.24	0.11–0.53	< 0.01	0.83	0.41–1.70	0.62	1.74	0.77–3.91	0.17	0.93	0.31–2.74	0.90	0.64	0.31–1.35	0.25
**Respondents’ monthly income (in PKR)**															
Up to 50,000	1			1			1			1			1		
> 50,000–100,000	2.15	1.09–4.23	0.02	0.67	0.33–1.34	0.25	1.45	0.71–2.98	0.31	0.51	0.21–1.30	0.16	1.96	1.00–3.85	0.05
> 100,000–150,000	1.10	0.51–2.33	0.80	0.84	0.38–1.85	0.67	1.12	0.49–2.55	0.77	0.77	0.27–2.13	0.61	2.23	1.02–4.85	0.04
> 150,000–200,000	0.47	0.19–1.19	0.11	0.44	0.17–1.11	0.08	1.52	0.59–3.92	0.38	1.92	0.66–5.57	0.23	2.65	1.01–6.94	0.04
> 200,000	0.83	0.35–1.96	0.67	0.28	0.11–0.69	0.01	2.11	0.85–5.19	0.10	1.38	0.47–4.03	0.54	1.37	0.57–3.26	0.47
**Age of teens**															
13–15 years	1			1			1			1			1		
16–17 years	0.34	0.19–0.58	< 0.01	0.79	0.47–1.33	0.38	1.26	0.74–2.15	0.39	0.67	0.33–1.38	0.28	0.59	0.34–1.03	0.06
18–19 years	0.25	0.15–0.44	< 0.01	1.07	0.64–1.79	0.77	1.11	0.65–1.88	0.69	0.62	0.30–1.26	0.18	0.51	0.29–0.87	0.01
**Sex of teens**															
Male	1			1			1			1			1		
Female	1.80	1.16–2.79	0.01	0.86	0.56–1.32	0.49	0.90	0.58–1.39	0.64	0.83	0.46–1.50	0.54	1.41	0.90–2.21	0.13
**Experience of teens’ internet addiction**															
No addiction	1			1			1			1			1		
Medium addiction	0.28	0.14–0.57	< 0.01	0.54	0.26–1.07	0.07	2.28	1.06–4.90	0.03	1.42	0.62–3.21	0.40	0.46	0.23–0.91	0.02
High addiction	0.56	0.31–1.01	0.05	3.07	1.77–5.32	< 0.01	3.18	1.67–6.07	< 0.01	0.63	0.30–1.30	0.21	1.12	0.63–1.99	0.68
**Respondents’ digital skills**															
Weak skills	1			1			1			1			1		
Good skills	3.41	2.12–5.51	< 0.01	1.58	1.01–2.47	0.04	1.94	1.20–3.15	0.01	0.95	0.51–1.75	0.87	4.05	2.52–6.49	< 0.01
**Threat appraisal**															
**Severity of online risks**															
Not serious	1			1			1			1			1		
Serious	3.87	1.84–9.16	< 0.01	1.32	0.66–2.64	0.43	1.52	0.71–3.27	0.28	1.13	0.42–3.05	0.81	2.99	1.48–6.06	< 0.01
**Susceptibility to online risks**															
Not likely	1			1			1			1			1		
Likely	1.56	1.01–2.41	0.04	0.91	0.59–1.41	0.67	1.06	0.67–1.65	0.81	2.06	1.07–3.95	0.03	1.29	0.83–2.03	0.26
**Coping appraisal**															
**Response-efficacy to perform online protection**															
Disagree	1			1			1			1			1		
Agree	0.33	0.14–0.73	0.01	0.94	0.43–2.05	0.88	2.17	0.96–4.89	0.06	1.57	0.57–4.34	0.38	0.43	0.19–0.96	0.04
**Self-efficacy to perform online protection**															
Not likely	1			1			1			1			1		
Likely	0.98	0.45–2.14	0.97	1.74	0.81–3.73	0.15	1.01	0.46–2.21	0.97	0.68	0.26–1.83	0.45	1.68	0.77–3.68	0.19
**Teens’ self-esteem**															
Low self-esteem	1			1			1			1			1		
High self-esteem	2.01	1.29–3.12	< 0.01	1.64	1.06–2.53	0.02	2.09	1.31–3.34	< 0.01	1.15	0.63–2.11	0.64	2.34	1.49–3.69	< 0.01
**Teens’ resilience**															
Low resilience	1			1			1			1			1		
High resilience	1.42	0.88–2.27	0.14	1.16	0.73–1.85	0.53	1.74	1.04–2.90	0.03	1.27	0.64–2.49	0.48	2.59	1.60–4.19	< 0.01

### Multivariate logistic regression analysis

Table 3 shows the multivariate logistic regression analysis presenting five models, one for each of the parental internet mediation dimensions and its predictors. The findings of Model I revealed that the respondents were more likely to apply active co-use mediation if they had female teens (AOR = 1.89, 95% CI: 1.03–3.51), had good digital skills (AOR = 2.53, 95% CI: 1.35–4.76), found the online risks for their teens serious (AOR = 3.42, 95% CI: 1.28–9.11) and judged their teen’s self-esteem to be high (AOR = 2.35, 95% CI: 1.27–4.32). On the other hand, applying active co-use mediation was less likely for the respondents whose children had medium internet addiction (AOR = 0.37, 95% CI: 0.14–1.02) compared to no addiction and who felt confident about their teens’ response-efficacy to perform online protection (AOR = 0.34, 95% CI: 0.12–1.03).

**Table 3:** Multivariate logistics regression of parental internet mediation with various socio-demographic and PM predictors

Characteristics	Model I Active co-use			Model II Restrictive			Model III Monitoring			Model IV Technical			Model V Active internet safety		
	AOR	95% CI	p-value	AOR	95% CI	***p***-value	AOR	95% CI	p-value	AOR	95% CI	***p***-value	AOR	95% CI	p-value
**Respondents’ age**															
31–40 years	1			1			1			1			1		
41–50 years	0.93	0.37–2.35	0.88	1.07	0.39–2.91	0.89	1.87	0.73–4.83	0.19	2.31	0.57–9.31	0.24	2.73	0.91–8.23	0.07
51–60 years	0.59	0.20–1.78	0.36	0.99	0.31–3.18	0.99	1.19	0.38–3.67	0.75	1.47	0.28–7.53	0.64	1.64	0.47–5.79	0.44
**Respondents’ monthly income (in PKR)**															
Up to 50,000	1			1			1			1			1		
> 50,000–100,000	2.22	0.89–5.51	0.10	0.35	0.13–0.92	0.03	0.95	0.40–2.25	0.91	0.55	0.17–1.74	0.31	1.57	0.63–3.90	0.32
> 100,000–150,000	0.94	0.35–2.57	0.83	0.63	0.21–1.86	0.40	0.66	0.25–1.73	0.39	1.08	0.31–3.75	0.89	2.55	0.87–7.47	0.08
> 150,000–200,000	0.38	0.11–1.29	0.09	0.16	0.04–0.57	< 0.01	0.82	0.26–2.59	0.73	4.03	0.98–16.5	0.05	2.21	0.61–8.02	0.23
> 200,000	0.93	0.28–3.08	0.96	0.14	0.04–0.51	< 0.01	1.58	0.52–4.83	0.42	3.28	0.78–13.7	0.10	1.57	0.46–5.36	0.47
**Age of teens**															
13–15 years	1			1			1			1			1		
16–17 years	0.60	0.24–1.47	0.26	0.26	0.10–0.65	< 0.01	0.54	0.24–1.21	0.13	0.31	0.10–0.92	0.03	0.31	0.12–0.80	0.01
18–19 years	0.42	0.15–1.18	0.09	0.23	0.07–0.68	0.01	0.39	0.15–0.99	0.04	0.33	0.09–1.12	0.07	0.17	0.05–0.54	< 0.01
**Sex of teens**															
Male	1			1			1			1			1		
Female	1.89	1.03–3.51	0.04	1.30	0.70–2.42	0.41	1.11	0.63–1.96	0.71	0.85	0.39–1.86	0.69	1.56	0.83–2.96	0.17
**Experience of teens’ internet addiction**															
No addiction	1			1			1			1			1		
High addiction	1.28	0.49–3.28	0.61	2.85	1.16–7.02	0.02	4.79	2.33–9.86	< 0.01	0.74	0.46–4.79	0.63	1.28	0.47–3.49	0.62
Medium addiction	0.37	0.14–1.02	0.05	0.32	0.13–0.83	0.01	1.48	0.57–3.81	0.08	1.49	0.42–2.54	0.50	0.23	0.08–0.67	< 0.01
**Respondents’ digital skills**															
Weak skills	1			1			1			1			1		
Good skills	2.53	1.35–4.76	< 0.01	1.59	0.84–3.01	0.15	2.02	1.11–3.68	0.02	0.54	0.24–1.25	1.51	1.94	1.03–3.67	< 0.01
**Threat appraisal**															
**Severity of online risks**															
Not serious	1			1			1			1			1		
Serious	3.42	1.28–9.11	0.01	1.50	0.58–3.83	0.39	1.29	0.53–3.13	0.57	0.97	0.24–3.94	0.96	2.24	0.86–5.78	0.09
**Susceptibility to online risks**															
Not likely	1			1			1			1			1		
Likely	1.08	0.58–2.00	0.80	0.87	0.47–1.64	0.68	0.88	0.50–1.56	0.67	2.54	1.07–5.99	0.03	0.91	0.47–1.73	0.77
**Coping appraisal**															
**Response-efficacy to perform online protection**															
Disagree	1			1			1			1			1		
Agree	0.34	0.12–1.03	0.05	1.28	0.42–3.88	0.66	3.07	1.06–8.89	0.03	0.75	0.17–3.41	0.71	0.23	0.07–0.73	0.01
**Self-efficacy to perform online protection**															
Not likely	1			1			1			1			1		
Likely	1.86	0.64–5.39	0.25	2.49	0.85–7.33	0.09	0.70	0.26–1.91	0.48	2.48	0.57–10.8	0.23	4.59	1.52–13.8	< 0.01
**Teens’ self-esteem**															
Low self-esteem	1			1			1			1			1		
High self-esteem	2.35	1.27–4.32	< 0.01	1.40	0.77–2.55	0.26	1.81	1.02–3.19	0.04	1.22	0.55–2.67	0.63	2.06	1.20–3.53	< 0.01
**Teens’ resilience**															
Low resilience	1			1			1			1			1		
High resilience	1.13	0.56–2.25	0.73	1.11	0.56–2.18	0.77	1.31	0.68–2.53	0.41	1.17	0.47–2.89	0.73	2.65	1.46–4.82	< 0.01

The results of Model II reveal that the respondents with higher incomes were generally less likely to apply restrictive mediation. Furthermore, those respondents with teens of older ages were less likely to apply restrictive mediation. High internet addiction – compared to no addiction – was a significant predictor for applying restrictive mediation (AOR = 2.85, 95% CI: 1.16–7.02), whereas parents were less likely to use this dimension of mediation for medium addiction (AOR = 0.32, 95% CI: 0.13–0.83).

Model III demonstrates that the adjusted odds of monitoring were lower for the respondents having teens between 18 and 19 years of age (AOR = 0.39, 95% CI: 0.15–0.99) compared to 13 and 15 years. The use of monitoring as a mediation dimension was more likely in cases of high internet addiction (AOR = 4.79, 95% CI: 2.33–9.86), for respondents with good digital skills (AOR = 2.02, 95% CI: 1.11–3.68), and for those caregivers who felt confident about their teens’ response-efficacy (AOR = 3.07, 95% CI: 1.06–8.89) and attributed high self-esteem to their children (AOR = 1.81, 95% CI: 1.02–3.19).

Model IV was not very clear because most of the predictors for technical mediation were not significant. However, caregivers who judged their teens’ susceptibility to online risks as likely were also more likely to use technical mediation (AOR = 2.54, 95% CI: 1.07–5.99).

The findings of Model V reveal a positive association between the use of active mediation of internet safety and good digital skills (AOR = 1.94, 95% CI: 1.03–3.67), self-efficacy to perform online protection (AOR = 4.59, 95% CI: 1.52–13.8), and considering high self-esteem (AOR = 2.06, 95% CI: 1.20–3.53) and resilience (AOR = 2.65, 95% CI: 1.46–4.82) among teens. The respondents were less likely to apply this kind of mediation when their teens were older than 13–15 years (16–17 years: AOR = 0.31, 95% CI: 0.12–0.80; 18–19 years: AOR = 0.17, 95% CI: 0.05–0.54), had a medium addiction to the internet (AOR = 0.23, 95% CI: 0.08–0.67) compared to no addiction, and if caregivers felt confident about their teens’ response-efficacy (AOR = 0.23, 95% CI: 0.07–0.73).

## Discussion

This study aimed to assess the various dimensions of parental internet mediation to regulate teens’ internet use and its predictors. Given the research objectives, the findings are based on parents’ perceptions and assessment about their teens’ online engagements and other dimensions, corresponding to previous studies [36, 37, 49, 50]. The research provides local evidence for Pakistan and, thus, adds value by filling some of the gaps in the existing literature.

The teens’ use of the internet on various portable devices, such as smartphones, laptops and tablets, increases their probability of internet addiction [51]. Moreover, the popularity of multiple apps and social networking sites, with the provision of instant notifications, also provoked the teens’ online addiction. The analysis found a medium to high level of internet addiction among teens, where the absence of smartphones and the internet caused anxiety. These results are similar to those of previous research conducted in Europe, Singapore and Pakistan [41, 52, 53].

The teens’ online activities are individualised and privatised, due to the variety of portable devices available and the multiple locations (homes, schools/colleges) where they are used. Hence, it is challenging for parents to oversee their teens [19]. Therefore, parental internet mediation is highly significant in guiding, supervising and regulating teens’ use of the internet. The results showed that parents in the district of Lahore applied mixed forms of mediation, most commonly the active mediation of internet safety, active co-use, restrictive and monitoring mediation; only a few parents adopted technical or software-related mediation. These findings are consistent with previous studies [20, 33, 54]. However, the results were slightly inconsistent compared to preceding research, which could be attributed to cultural variations. Parents in the United States, for example, preferred active co-use mediation [55], whilst they applied both active and restrictive mediation in Europe [56]. Our findings demonstrated that parents in Pakistan generally preferred to apply a combination of mediation according to their own priorities and values as well as their teens’ needs and competences [20]. This has also been argued in other studies, where a mixed approach was found to be the most effective [17, 57].

This research also featured the role of parental threat appraisal and coping appraisal in determining their preference for various mediation strategies. This study demonstrates that parents found online risks to be serious and judged it likely that their teens would be susceptible. Furthermore, a majority of respondents also endorsed their teens’ self- and response-efficacy to ensure online safety in the face of risks. Similar results are also evident in past studies, highlighting the significance of parental threat and coping appraisal to mediation [23]. Furthermore, the results also indicate that those parents who reported high self-esteem amongst their teens were more likely to apply active co-use, internet safety mediation and monitoring. These findings somewhat corroborate previous research [58]. Lastly, the findings highlight that the respondents applied active internet safety mediation when they found high resilience among their teens. This research conceptualises resilience as a positive outcome of parental internet mediation for coping with risks. The results also suggested that parental guidance and certain individual traits, including improved coping skills and high self-esteem, are essential for nurturing resilience [25].

Summarising the above, this research highlights the parental perception and infers that parental internet mediation influences critical thinking and resilience in teens to minimise online risks and maximise opportunities. Since resilience and risks go hand in hand, the findings support the recommendation that parental internet mediation enables teens to cope with adverse situations as a strength-based approach.

### Limitations

No causal relationships can be drawn due to the cross-sectional study design. One of the key limitations could be regarding the data obtained, highlighting only parents’ perception, particularly about their teens’ dimensions. However, this methodological approach has been widely used and adopted in various studies. A varied analytical approach was adopted, after performing necessary statistical tests to assure reliability and avoid any errors or outliers, due to skewed data for some variables, which may have had an undue influence on the statistical analysis [59, 60]. Another limitation was faced during the fieldwork regarding interviewing eligible respondents at their workplaces. Therefore, the researchers initially contacted the relevant authorities at each institution sampled, briefed them on the research objective and requested a permission letter from them to support access. Thus, eligible respondents were approached and an appointment was scheduled for the interview. However, this might also have enforced a selection bias as people with no or very low activity in internet mediation may not have participated.

### Policy and practical implications

This research highlighted the significance of parental internet mediation and multiple predictors. The research reiterated that the role of parents is critical in regulating teens’ use of internet, keeping in view their day-to-day activities and personal dimensions, such as self-esteem and resilience. Parents regulate and guide their teens about the appropriate use of the internet, enabling them to critically analyse the situation and adopt optimised online behaviour while in the face of risks. The local evidence generated through this research facilitates the parents, researchers and educators to realise the increasing online engagement of teens and the role and benefits of parental internet mediation. The findings also drew the attention of researchers and policymakers to develop, adapt and implement e-safety guidelines and resource material to ensure the online safety of young generations. There is a need for organised awareness creating programmes at schools/colleges and community levels for both teens and parents regarding cyber risks and safety behaviours. Practitioners working with and for both parents and teens can benefit from the insights of this research, particularly in understanding how PM facilitates the resilience among teens.

## Conclusion

The researchers conclude that parental internet mediation is a multidimensional concept which is directed towards not only regulating teens’ use of the internet but also augmenting their abilities to create resilient pathways to prevent online risks. Therefore, it is necessary to implement PM guidelines, e-safety resource material and local support networks to raise community awareness and promote positive outcomes among teens. Based on parents’ perceptions, these findings also support the suggestion of launching government-supported initiatives and updating the curriculum module to raise awareness among parents, teachers, professionals and communities about potential online risks, online protection tools and safer internet best practices in order to cultivate a safe environment for children, teens and the young generation. It also highlights the social responsibility of internet service providers to block offensive and hate-filled websites/pages. Lastly, the research emphasises the need to initiate community-based programmes to educate parents, teachers and teens about online safety tools and mechanisms.

## Supplementary Information

**Additional file 1: Supplementary Appendix 1**: Questionnaire

**Additional file 2: Supplementary Appendix 2**: Operationalization of variables – Details of the scales/indexes

## Data Availability

Data are available from authors upon reasonable request.

## Abbreviations

AOR: Adjusted odds ratio
CI: Confidence interval
OR: Odds ratio
PKR: Pakistani rupee
PM: Parental mediation
PMT: Protection Motivation Theory
SD: Standard deviation
SPSS: Statistical Package for the Social Sciences
