# Supplementary material for: Predictors of parental mediation in teenagers’ internet use: a cross-sectional study of female caregivers in Lahore, Pakistan
Source: BMC Public Health. 2021 Feb 8;21:317. doi: 10.1186/s12889-021-10349-z (PMC7871392; doi:10.1186/s12889-021-10349-z)
Supplement: Supplementary file 1 — Additional file 1: Supplementary Appendix 1: Questionnaire [file 12889_2021_10349_MOESM1_ESM.pdf]

Supplementary Appendix 1: Questionnaire

Parental Mediation in Internet Use and Resilience among their Teenagers

Interviewer ID: ____________ **Questionnaire ID: _____________**

Sr. #	Question	Response	Yes	No
**1. Identification**				
1.1	Type of Institution/Facility:	1. Medical 2. Non-Medical		
1.2	Name of Institution/Facility	____		
1.3	Name of department	____		
**2. Socio-Demographic Characteristics**				
2.1	What is your age?	_________Years *(Age in completed years)*		
2.2	What is yours current marital status? *(Encircle the relevant response)*	1. Married<br>2. Separated<br>3. Divorced<br>4. Widowed		
2.3	What is your monthly Income?	Rs. _________________		
2.4	Do you have a teen between 13-19 years?	1. Yes<br>2. No *(End the Interview)*		
2.5	How old your youngest teen is?	________Years (Age in completed years)		
2.6	What is sex of your youngest teen?	1. Male<br>2. Female		
**3. Perception about Teen's Internet use**				
3.1	Does your teen use internet?	1. Yes<br>2. No		
3.2	Do these things ever happen to your teen?		**Yes**	**No**
		1. Gone without eating or sleeping because of internet	1	2
		2. Felt bothered when cannot be on internet	1	2
		3. Caught surfing when is not really interested	1	2
		4. Spent less time with family & friends because of internet	1	2
		5. Tried unsuccessfully to spend less time on internet	1	2
**4. Parental Internet Mediation**				
4.1	Which of these digital skills do you know how to do on internet?		**Yes**	**No**
		1. Compare different websites to decide if information is true	1	2
		2. Change filter preferences	1	2
		3. Bookmark a website	1	2
		4. Block pop ups/unwanted adverts or junk mail/spam	1	2
		5. Delete the record of which sites he/she has visited	1	2
		6. Change privacy setting on a social networking profile	1	2
		7. Block messages from someone don't want to hear to	1	2

Sr. #	Question	Response	Yes	No
4.2	Do you mediate in teen use of internet through following?		**Yes**	**No**
		1. Talk about what your teen does on the internet	1	2
		2. Encourage teen to explore and learn things on the internet	1	2
		3. Sit with your teen while he/she uses the internet	1	2
		4. Stay nearby when your teen uses the internet	1	2
		5. Share activities together with your teen on the internet	1	2
4.3	Do you restrict or allow your teen with your permission for these things?		**Yes**	**No**
		1. Has own social networking profile	1	2
		2. Share personal information with others on the internet	1	2
		3. Use instant messaging (e.g., MSN, WhatsApp, Skype)	1	2
		4. Download music or films on the internet	1	2
		5. Watch video clips on the internet	1	2
		6. Upload photos, videos or music to share with others		
4.4	After teenager' use of internet, do you check/monitor the following?		**Yes**	**No**
		1. Which websites visited	1	2
		2. Which friends added to social network profile/instant messaging	1	2
		3. Messages in teen' email or instant messaging account	1	2
		4. Teen profile on a social networking or online community	1	2
4.5	Do you mediate your teen by any of these mechanisms?		**Yes**	**No**
		1. Parental controls or other means of blocking/filtering some websites	1	2
		2. Parental controls or other means of keeping track of visited websites	1	2
		3. A service or contract that limits the time teen spend on internet	1	2
		4. Software to prevent spam or junk mail/viruses	1	2
4.6	Do you mediate to ensure teen's internet safety through?		**Yes**	**No**
		1. Help when teen find something difficult to do on internet	1	2
		2. Suggest ways to use the internet safely	1	2
		3. Explain why some websites are good or bad	1	2
		4. Suggest ways to behave towards other people online	1	2
		5. Talk to teen about what to do if something on internet bothers	1	2
		6. Helped in past when something has bothered your teen online	1	2

Sr. #	Question	Response	
**5. Predictors of Parental internet Mediation**			
**5.1**	**How serious do you find these online risks to your teen?**	**Serious**	**Not Serious**
5.1.1	If being threatened online**?**	1	2
5.1.2	If receive hate emails?	1	2
5.1.3	If receive unpleasant sexual remarks online?	1	2
5.1.4	If someone pretends to be your teen online?	1	2
5.1.5	If someone publishes teen' personal information online with bad intentions?	1	2
5.1.6	If someone posts teen' personal photos or videos online to harm him/her?	1	2
5.1.7	If someone posts negative rumors/inflammatory remarks about teen online?	1	2
5.1.8	If receive computer viruses, intentionally sent to teen?	1	2
**5.2**	**How likely do you feel, these risks may happen to your teen?**	**Likely**	**Not Likely**
5.2.1	If being threatened online**?**	1	2
5.2.2	If receive hate emails?	1	2
5.2.3	If receive unpleasant sexual remarks online?	1	2
5.2.4	If someone pretends to be your teen online?	1	2
5.2.5	If someone publishes teen' personal information online with bad intentions?	1	2
5.2.6	If someone posts teen' personal photos or videos online to harm him/her?	1	2
5.2.7	If someone posts negative rumors/inflammatory remarks about teen online?	1	2
**5.3**	**To what extent do you agree that your teen adopt these online protection behaviors?**	**Agree**	**Disagree**
5.3.1	Does teen know how to use nicknames to mask identity online?	1	2
5.3.2	Does teen know how to protect personal information online?	1	2
5.3.3	Does teen know when to provide inaccurate information to protect privacy?	1	2
5.3.4	Does teen know how to limit access to personal information online for friends and family only?	1	2
5.3.5	Does teen know how to avoid strangers online who may harm him/her?	1	2
5.3.6	Does teen know whom to talk to for advice for online protection?	1	2
5.3.7	Does teen realize that talking to parents or teachers will help in getting good advice for online protection?		
**5.4**	**How likely do you feel, your teen is confident to perform these protection behaviors?**	**Likely**	**Not Likely**
5.4.1	Does teen know how to use nicknames to mask identity online?	1	2
5.4.2	Does teen know how to protect personal information online?	1	2
5.4.3	Does teen know when to provide inaccurate information to protect privacy?	1	2
5.4.4	Does teen know how to limit access to personal information online for friends and family only?	1	2
5.4.5	Does teen know how to avoid strangers online who may harm him/her?	1	2
5.4.6	Does teen know whom to talk to for advice for online protection?	1	2

Sr. #	Question	Response	
**6. Perception about Teens' self-esteem and resilience**			
**6.1**	**How true do these statements, represent the general feelings of your teen?**	**Yes**	**No**
6.1.1	Is teen satisfied with him/herself?	1	2
6.1.2	Does feel that teen is a person of worth (on an equal level with others)?	1	2
6.1.3	Is feel useless at times?	1	2
6.1.4	Does feel that teen has a number of good qualities?	1	2
6.1.5	Is inclined to feel that he/she is a failure (not good at all)?	1	2
6.1.6	Is able to do things like most other people?	1	2
6.1.7	Is feel that teen doesn't have much to be proud of?	1	2
6.1.8	Does take a positive attitude toward him/herself?	1	2
6.1.9	Has wish that teen could have more respect for him/herself?	1	2
6.1.10	at times, he/she thinks that he/she is not good at all		
**6.2**	**To what extent do the following describe your teen' resilient behavior?**	**Yes**	**No**
6.2.1	Has people around teen, wants to be live with?	1	2
6.2.2	Does know where to go to get help?	1	2
6.2.3	Is getting an education important to teen?	1	2
6.2.4	Is feel of belonging at teen' school/college/university?	1	2
6.2.5	Is try to finish what teen starts?	1	2
6.2.6	Has chances to learn things that will be useful for teen when grow older (e.g. cooking, working, helping others)?	1	2
6.2.7	When things don't go teen's way, can fix it without hurting anyone or him/herself?	1	2
6.2.8	Do you know a lot about teen (e.g. what makes happy, sad)?	1	2
6.2.9	Do you and your family care about teen when times are hard (e.g. if teen is sick or has done something wrong)?	1	2
6.2.10	Do teen's friends care about him/her when times are hard?	1	2
6.2.11	Does treat fairly in his/her community?	1	2
6.2.12	Does like the way teen' community celebrates holidays and festivals?	1	2

Thank you for your kind response and time.
