# Supplementary material for: Predictors of parental mediation in teenagers’ internet use: a cross-sectional study of female caregivers in Lahore, Pakistan
Source: BMC Public Health. 2021 Feb 8;21:317. doi: 10.1186/s12889-021-10349-z (PMC7871392; doi:10.1186/s12889-021-10349-z)
Supplement: Supplementary file 2 — Additional file 2: Supplementary Appendix 2: Operationalization of variables – Details of the scales/indexes [file 12889_2021_10349_MOESM2_ESM.pdf]

Supplementary Appendix 2:
Operationalization of variables – Details of the scales/indexes

1) **Parental internet mediation dimensions**

a) Active mediation

Talk about what teen does on internet

Encourage teen to learn and explore on internet

Sit with teen, while he/she uses internet

Stays nearby, when teen uses internet

Share activities together with teen on internet

b) Restrictive mediation

Has own social networking profile

Share personal information on internet

Use instant messaging (e.g. WhatsApp)

Download films/music online

Watch video clips online

Upload pictures, music or videos to share with others

c) Monitoring

Which websites visited

Which friends added to instant messaging/SNS profile

Messages in email or SNS accounts

Profile on SNS or online community

d) Technical mediation

Parental control means to block/filter some websites

Parental control means to keep track of visited websites

A service to limit teens' online screen time

Software to prevent viruses, junk mail/spam

e) Active mediation of internet safety

Help teen when find something difficult online

Suggest internet safely measures/ways

Explain good or bad websites

Recommend behavior towards other people online

Talk/discuss what to do if something bothers online

Helped teen in past, when bothered online

2) Respondents' digital skills

Compare different websites

Change filter settings

Bookmark a website

Block unwanted adverts/pop-ups or junk mail/spam

Delete record of visited websites

Change privacy settings on SNS

Block messages

3) Experience of teens' internet addictions

Don't eat or sleep because of internet

Feel bothered when can't be online

Caught surfing when really not interested

Spent less time with family and friends because of internet

Tried to spend less time online unsuccessfully

4) Threat appraisal

a) Severity of online risks to teen

Being threatened online

Receive hate emails

Receive unpleasant/sexual remarks online

Someone pretends to be your teen online

Someone publishes teen' personal information online with bad intentions

Someone posts teen' personal photos or videos online to harm him/her

Someone posts negative rumors/inflammatory remarks about teen online

Receive computer viruses, intentionally sent to teen

b) Susceptibility of teens' to online risks

Being threatened online

Receive hate emails

Receive unpleasant/sexual remarks online

Someone pretends to be your teen online

Someone publishes teen’ personal information online with bad intentions

Someone posts teen’ personal photos or videos online to harm him/her

Someone posts negative rumors/inflammatory remarks about teen online

5) Coping appraisal

a) Response-efficacy to perform online protection behaviors

Knows how to mask identity online

Knows how to protect personal information online

Knows when to provide inaccurate information online for privacy protection

Knows how to limit access online for friends/family

Knows how to avoid strangers online

Knows whom to talk to for advice of online protection

Realize that talking to parents/teachers help in getting good advice

b) Self-efficacy to perform online protection behaviors

Knows how to mask identity online

Knows how to protect personal information online

Knows when to provide inaccurate information online for privacy protection

Knows how to limit access online for friends/family

Knows how to avoid strangers online

Knows whom to talk to for advice of online protection

6) Self-esteem

On the whole, satisfied with him/herself

At times, thinks that he/she is not good at all*

Feels that he/she has a number of good qualities

Able to do things like most other people

Feels that he/she doesn’t have much to be proud of*

Certainly feels useless at times*

Feels that he/she is a person of worth

Wishes that he/she could have more respect for him/herself*

Inclined to feel that he/she is a failure*

Takes a positive attitude toward him/herself

7) Resilience

Has people around teen, wants to be live with

Does know where to go to get help

Is getting an education important to teen

Is feel of belonging at teen’ school/college/university

Is try to finish what teen starts

Has chances to learn things that will be useful for teen when grow older (e.g. cooking, working, helping others)

When things don’t go teen’s way, can fix it without hurting anyone or him/herself

Do you know a lot about teen (e.g. what makes happy, sad)

Do you and your family care about teen when times are hard (e.g. if teen is sick or has done something wrong)

Do teen’s friends care about him/her when times are hard

Does treat fairly in his/her community

Does like the way teen’ community celebrates holidays and festivals

These items were reverse coded
